# Two-Stage Revision TKA Is Associated with High Complication and Failure Rates

**DOI:** 10.1155/2014/659047

**Published:** 2014-12-24

**Authors:** Christopher E. Pelt, Ray Grijalva, Lucas Anderson, Mike B. Anderson, Jill Erickson, Christopher L. Peters

**Affiliations:** Department of Orthopaedic Surgery, University of Utah, 590 Wakara Way, Salt Lake City, UT 84108, USA

## Abstract

Despite two-stage revision remaining the gold standard in treating periprosthetic infection of total knee arthroplasty (TKA), there remains uncertainty regarding the actual success rate and the risk factors for failure. We retrospectively reviewed 58 knees with mean follow-up of 38 months who underwent two-stage revision TKAs from 1998 to 2012 by a single surgeon. Failure was defined as persistent infection or reoperation after two-stage revision TKA surgery. Failure occurred in 36%. The overall mortality was 22%. The mean time to reinfection was 26 months. Polymicrobial infection was associated with a higher risk of failure (RR 3.31, *P* < 0.001). Knees requiring soft tissue coverage were also at a greater risk of failure (RR 2.67, *P* = 0.001), as were knees that underwent four or more additional surgeries after the primary TKA and prior to stage-one explantation (RR 2.25, *P* = 0.020). Thus, opportunities exist for improvement in management of infected TKA.

## 1. Introduction

Periprosthetic joint infection (PJI) remains a devastating and complicated problem in the joint replacement population with an incidence of 1–3% [1–3]. Treatment options for periprosthetic infections range from debridement with retention to single-stage revision or two-stage revision. Currently, two-stage revision remains the gold standard for treatment of TKA infection in the United States [4–11]. Although this treatment regimen has increased, failures persist. The cause and nature of these failures are not well understood, giving clinicians little to go on when predicting which knee infections are most likely to fail a two-stage revision procedure. Because of this, recent studies have examined the clinical factors associated with failed two-stage revision [9, 12–17]. These studies have shown an association with increased operative time during reimplantation [14], needing additional interval surgeries between stages and the presence of lymphedema on physical exam to be predictive of failure [18]. Previous reports have also shown that methicillin-resistant* Staphylococcus aureus* (MRSA) and culture negative infections have a higher rate of two-stage revision failure [14, 17].

Although these previous studies have shed light on this issue, there remains an incomplete understanding of factors associated with failure, the eradication rate, and the complications of the two-stage approach. The purpose of this study was to report the results and complications of two-stage revision TKA for the treatment of PJI and identify factors associated with failure.

## 2. Materials and Methods

**Figure 1:**
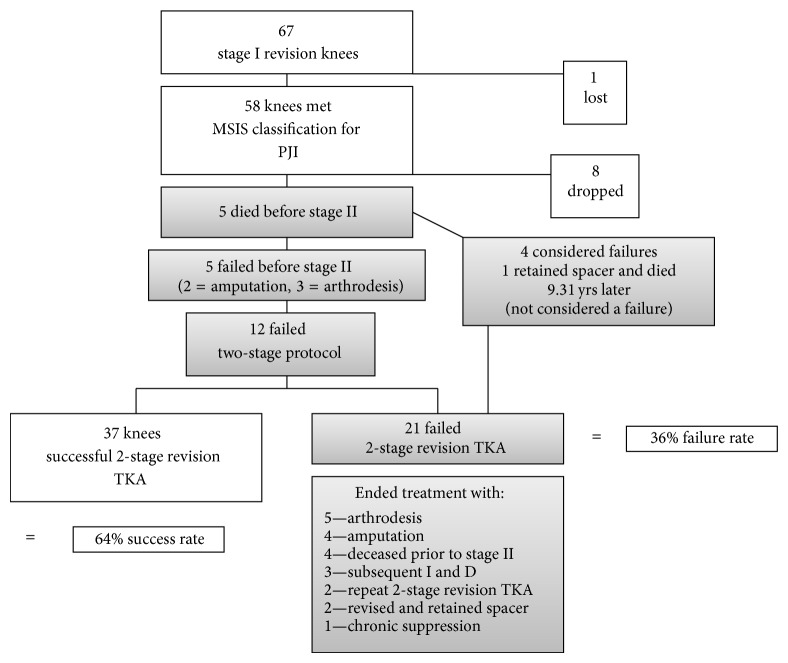
Flow diagram demonstrating the inclusion and exclusion of participants in the analysis.

After institutional review board approval, we queried the surgical database of our academic medical center for patients who underwent a stage-one explantation and antibiotic spacer placement for the treatment of PJI from 1998 to 2010 by a single surgeon. We identified 67 TKAs that underwent stage-one explantation and antibiotic spacer placement, all with the intent to proceed to later stage-two reimplantation (Figure 1). One patient left the country after being discharged and was lost to follow-up. PJI was diagnosed according to the classification established by the Musculoskeletal Infection Society (MSIS) [26]. Eight patients did not meet the MSIS classification of PJI and were removed from the analysis. This resulted in 56 patients (58 knees) available for review. Demographics were recorded (Table 1). Of the 58 knees, 69% (40/58) originally underwent primary TKA due to osteoarthritis, 12% (7/58) for rheumatoid arthritis, and 12% (7/58) for posttraumatic arthritis and in 7% (4/58) we were unable to identify the reason for the primary diagnosis. Ten knees (17%) presented to clinic with either an abscess or soft tissue damage prior to stage one. Eight (14%) of these required consulting with plastic surgery for planned flap repair.

The surgical protocol consisted of a two-stage approach. Stage one involved irrigation and debridement of all nonviable tissue, radical synovectomy, and removal of all implants and cement. All patients then received one of three types of an antibiotic spacer based upon surgeon preference at the time of the operation. The vast majority received a new cobalt-chrome primary femoral component (Maxim or Vanguard, Biomet Inc., Warsaw, IN, USA) and a new cemented polyethylene tibial bearing of either a cruciate retaining or anterior stabilized design (Biomet, Warsaw, IN, USA), without the modular baseplate. Other spacers included all cement molded articulating spacers or static cement blocks. Polymethylmethacrylate (PMMA) cement was used for fixation (Palacos, Zimmer, Warsaw, IN, USA; or Cobalt, Biomet Inc., Warsaw, IN, USA) to which additional antibiotics were added, including a combination of tobramycin (1.2–3.6 g per batch of cement), vancomycin (500 mg–2 g per batch of cement), cefuroxime (750 mg per batch of cement), and/or gentamycin (500 mg per batch of cement). Cement was mixed and then allowed to set to a doughy state while affixed to the implants prior to implantation to prevent aggressive interdigitation to the surrounding bone, thereby facilitating easier removal at planned stage-two reimplantation. Antibiotics and their dosages were occasionally adjusted based on preoperatively identified speciation of the organism. The patella was left unresurfaced after removal of the implant and cement. Monofilament suture was used for wound closure. Suction drainage was avoided to allow the antibiotic from the spacer to remain in the joint. A Bulky Jones dressing was applied, and patients were mobilized under the guidance of in-hospital physical therapists starting on postoperative day one with 50% protected weight bearing and assistance device use at all times. Additionally, if patients had soft tissue damage that required additional coverage, a plastic surgeon was consulted to perform a gastrocnemius flap at the conclusion of stage one. All patients were treated with parenteral antibiotics and some received oral rifampin in addition, for a minimum of six weeks, targeted to organism sensitivity. This was followed by a four- to six-week antibiotic holiday to allow time for any potential regrowth. If infection markers normalized then patients proceeded to stage two at the planned 3-month interval. Otherwise further treatment to eradicate the organism was performed. Chronic oral suppression was not used in the treatment period but may have been included as an option for long-term treatment after stage two. The second stage consisted of removal of the spacer and reimplantation with all new components (Maxim or Vanguard, Biomet Inc., Warsaw, IN, USA).

Failure was defined as persistent infection or reoperation after two-stage revision TKA surgery. Variables evaluated for failure were prospectively collected and include gender, age, BMI, ASA score, type of stage I spacer, smoking, simultaneous soft tissue coverage, type of organism, additional surgeries prior to stage one, additional surgeries prior to stage-two reimplantation, and type of infection. Infections were classified as early, delayed, or late based upon the recommendations by Zimmerli et al. [27]. Additionally, the number and type of complications associated with stage one were determined. Mortality was determined from chart review and by reviewing the Social Security Death Index.

Given that the failures occurred after the predictor variables were measured, the data came from a retrospective cohort study design. With this study design, incidence proportions could be calculated. Therefore the data were modeled using modified Poisson regression [28], which enabled reporting the data as risk ratios. A likelihood ratio test was performed to see if having two knees from one patient introduced enough lack of independence among the observations to require a mixed effects modeling approach, confirming that the data were sufficiently independent to assume all knees were independent observations. A *P* value of <0.05 was considered as significant. Data were analyzed using STATA V.13 (College Station, TX, USA).

## 3. Results

Of the 58 knees that met criteria, 48 (83%) completed the planned two-stage reimplantation, and ten patients (17%) were unable to go on to eventual stage-two reimplantation (Figure 1). Of those 58 knees, 37 (64%) were ultimately deemed successful without persistent infection after two-stage revision TKA. Thirty-six percent (21/58) of our sample had persistent or recurrent infection and were considered as having failed two-stage revision TKA. Of the ten knees that did not undergo a stage-two reimplantation, nine were included as failures. Five of these were for persistent infection and four were due to death prior to stage two. One patient elected not to undergo stage-two reimplantation due to adequate functional results with the articulating spacer and ultimately died nine years after stage one due to “natural causes” and this patient was not considered a failure. Of the four deaths considered failures, two had recurrent infections, one resolved with irrigation and debridement, and the other was recommended above knee amputation; the cause of death is unknown for these two. The third patient died on postoperative day three due to a myocardial infarction associated with ventricular tachycardia. The cause of death for the fourth patient is also unknown.

Seven knees (12%) required additional surgery after stage one and prior to stage-two reimplantation. Three (43%) underwent irrigation and debridement; three (43%) had a repeat stage one with spacer exchange and one knee (14%) was converted from an articulating spacer to a static spacer. Four of the seven went on to fail two-stage revision TKA (RR 1.71, 95% CI 0.80–3.65, *P* = 0.163). Aside from failure or death, there were 4 total complications prior to stage two (4/58, 7%), 2 cement mold fractures and 2 intraoperative tibia fractures, and these were not considered failures.

As mentioned, 21 of the 58 knees (36%) that underwent stage one were deemed failures due to persistent or recurrent infection and/or related reoperation. Ultimately these patients ended their treatment with arthrodesis, above knee amputation, repeat two-stage revision TKA, rerevision and retention of the spacer after repeat stage one, chronic suppression, or death (Figure 1). The mean time to reinfection following the initial stage-two procedure was 26 months (range 2–72 months), with 50% failing at 24 months or later. Following stage-two reimplantation, 21% (10/48) required another irrigation and debridement. The 90-day mortality was 1.7% (*n* = 1) (Table 2) and the mean time to death from stage one was 46 months (0–112 months).

Of those that failed, 6/21 (29%) showed a new organism at the time of failure and 5/21 (24%) showed the same organism. Four of the 21 (19%) were deceased prior to stage II and 4/21 (19%) had their initial organism eradicated; however 2 of these required subsequent reoperation (irrigation and debridement). In one knee (5%) the organism was unknown at the time of failure because the knee was revised elsewhere and culture results were not available and one other knee (5%) had culture negative results at failure (Table 3). Univariate analysis was performed to determine if certain types of organisms were associated with failure.* Streptococcus* demonstrated an increased risk for failure (Table 4); however, this organism was eradicated in all cases and different organisms resulted in the failure. Additionally, all knees with* Streptococcus* at stage 1 had multiple organisms present and those same patients had an increased risk for reinfection (RR 3.31, 95% CI 2.19–5.00, *P* < 0.001). Our data suggests no increased risk for infection with methicillin-sensitive* Staphylococcus aureus* (MSSA), MRSA, or coagulase negative* Staphylococcus* (all *P* > 0.77). With the numbers available we were unable to assess the potential risk for other organisms.

Poisson regression analysis for univariable data showed that simultaneous soft tissue coverage (*P* = 0.001) and four or more additional surgeries from the time of primary TKA to stage one (*P* < 0.020) were associated with failure (Table 5). We found no other patient characteristics or surgical factors to be associated with failure in this series. Interestingly, 39 knees had undergone an additional surgery between the primary TKA and stage one. Fourteen knees (24%) received a single irrigation and debridement (I&D) for expected infection, 9 knees (26%) underwent revision TKA for suspected infection, six (10%) underwent I&D with exchange of the polyethylene due to infection, five (9%) underwent one or more I&Ds as well as revision TKA for suspected infection, one knee (2%) had an extensor mechanism repair, another (2%) underwent I&D with soft tissue repair due to infection, one patient (2%) had revision TKA with patellectomy, and one knee (2%) underwent previous patellectomy. Additionally, one patient was revised prior to stage one for aseptic loosening. Four patients had four or more additional surgeries after primary TKA and before stage one.

## 4. Discussion

Although two-stage revision TKA, originally described by Insall et al. [7], has become the method of choice for revision TKA due to infection, failures persist. Our failure rate of 36% is higher than previously reported [29, 30]. However, our failure rate includes death prior to planned stage II, any reoperation following stage II, and failure to eradicate infection. A review of the literature revealed an average failure rate of 17.3% (Table 6) with failure rates ranging from 0 to 34%. We also found a 7% surgical complication rate associated with stage-one antibiotic spacers which consisted of two cement mold fractures and two intraoperative tibia fractures. Looking at factors associated with failure, our results show that polymicrobial infection, the need for soft tissue coverage, and the need for multiple (≥4) surgeries between primary TKA and stage one are associated with two-stage reimplantation failure.

There are limitations of the present study, including average follow-up of 38 months, which may still be insufficient to accurately detect the number of two-stage failures. Other limitations include those inherent to a retrospective review, though the follow-up of our cohort was quite good with only one patient lost after moving out of the country. In addition, there was a lack of standardization in both surgical protocol and postoperative medical management. Spacer types were left to the discretion of the treating surgeon, with a preference for the use of the functional articulating antibiotic spacer and all cement and static spacers more often used in complex cases (significant bone loss) or prior failures (rerevisions). Although different antibiotics and/or spacer types were used in this study, the treatments all followed well-established protocols. Antibiotics that were tailored to preidentified speciation of the infecting organism may have been used in lieu of a standard “one size fits all” recipe of the same antibiotics in every case (e.g., the addition of cefuroxime and lower dose of vancomycin in methicillin sensitive* Staphylococcus aureus*). Ultimately, during the retrospective nature of this study, which looks at the collection of seemingly different ingredients that all were used in the two-stage recipe to achieve the same product (eradication of infection), the present study represents a “real world” depiction of our experience with the two-stage approach to the treatment of periprosthetic infections in TKA, as opposed to the success or failure with any one particular spacer device, technique, or antibiotic dosage.

In Insall's original series of eleven patients all components were removed, without subsequent antibiotic spacer placement, and the patients underwent six weeks of parenteral antibiotic treatment before reimplantation [7]. There were no recurrent infections during the 34-month average follow-up period in that study. Other reports have not been as optimistic. In 1995 Hofmann et al. [31] reported on 26 patients treated with two-stage reimplantation with antibiotic spacer placement and also found no recurrent infections over a thirty-month average follow-up. Then a subsequent follow-up of these patients in 2005 showed a 12% reinfection rate occurring at average 35 months after reimplantation suggesting that the initial follow-up was too short [25].

With the available numbers, the present study did not show an association between resistant organisms and recurrent infection after two-stage reimplantation, though previous publications have shown both MRSA and other high virulence organisms to be associated with two-stage reimplantation failure [14, 17, 23]. High virulence organisms are known to produce adhesion factors, which not only allow bacteria to penetrate and remain within joint tissues making them difficult to eradicate, but also allow bacteria to penetrate soft tissues and cause more extensive destruction necessitating reconstructive procedures or additional surgeries [14]. We did, however, find that polymicrobial infections were associated with two-stage failure in the present study and may be due to antibiotic selection that is appropriate for one organism but not the other. In addition a different organism was found in 29% of our cases with reinfection suggesting that the initial culture results represent either a sampling error or the fact that the stage-one procedure introduced a new pathogen. These results highlight the importance of accurate pathogen identification and appropriate antibiotic selection in the treatment of chronic periprosthetic joint infection.

Two other risk factors for failure were demonstrated in the present study. Simultaneous soft tissue coverage procedure performed during stage-one revision was an independent risk factor for recurrent infection, a finding which may be consistent with previous reports showing an increased risk with prolonged operative time [14]. The operative time may be a surrogate of the increased exposure time to the outside environment or a more complex surgical site infection and deficiency of adequate soft tissues to facilitate infection eradication. Our results also demonstrated that having four or more surgeries prior to stage I was a risk factor for persistent infection after stage-two reimplantation.

In conclusion, chronic total knee arthroplasty infection remains difficult to treat. Although two-stage revision is considered the gold standard, there remains an incomplete understanding of the factors associated with failure. Polymicrobial infection, the need for soft tissue coverage procedures, and patients who underwent 4 or more additional surgeries prior to stage I were all associated with recurrent infection after two-stage reimplantation in the present study. Opportunities exist for improvement in the management of infected total knee arthroplasty including refinement of surgical and antibiotic protocols. Further research is needed to identify more modifiable risk factors associated with failure as well as prospective trials comparing the outcomes of one-stage revision TKA with two-stage revision TKA for the treatment of infected total knee arthroplasty.

## Figures and Tables

**Table 1:** Demographics.

Total sample, *N*	58 knees
Sex, *n* (%)	
Male	34 (59%)
Female	24 (41%)
Age (years), mean ± sd (range)	63 ± 11 (35–85)
BMI (kg/m^2^), mean ± sd (range)	33.4 ± 11.92 (15.90–79.52)
Patients with a BMI ≥35 kg/m^2^, *n* (%)	27 (47%)
ASA score ≥3, *n* (%)	17/58 (29%)
Follow-up (months), mean (range)	38 (0^*^–113)
Spacer type, *n* (%)	
Articulating antibiotic-loaded prosthesis	43 (75%)
Articulating or static all cement	14 (25%)
Immunocompromised patients, *n* (%)	3 (5%)
Proportion of patients with diabetes mellitus, *n* (%)	21 (36%)
Patients requiring dialysis, *n* (%)	4 (7%)
Patients who smoke, *n* (%)	7 (12%)

^*^One patient died 3 days after surgery.

**Table 2:** Mortality.

Time period	Rate
90-day mortality rate	1/58 (1.7%)
1-year mortality rate	2/58 (3%)
5-year mortality rate	9/58 (16%)
Overall mortality rate (mean TTD 4 yrs, 3 days to 10 years)	13/58 (22%)
Mortality prior to stage II	5/58 (8.6%)

**Table 3:** Bacteria present at time of initial surgery and at time of failure.

Initial organism	Organism at failure	New organism at failure
Coagulase negative *Staphylococcus *	Eradicated^1^	N/A
MSSA^2^	*Staphylococcus lugdunensis *	Yes
Gram + Cocci	Deceased	N/A
MRSA^3^	Deceased	N/A
Coagulase negative *Staphylococcus *	Unkown^4^	N/A
MSSA	Eradicated	N/A
Vancomycin resistant *Staphylococcus*, yeast, and *Streptococcus *	*Citrobacter braakii *	Yes
*Staphylococcus pneumoniae *	*Staphylococcus pneumoniae *	No
MSSA, bacteremia, and *Staphylococcus pneumoniae *	Deceased	N/A
MSSA	Enterobacter	Yes
MSSA	MSSA	No
Coagulase negative *Staphylococcus *	MRSA	Yes
MRSA, *Streptococcus *	MRSA	No
Coagulase negative *Staphylococcus*, *Acinetobacter*, *Streptococcus*, and *Pseudomonas *	*Candida albicans *	Yes
Coagulase negative *Staphylococcus *	Eradicated	N/A
Coagulase negative *Staphylococcus*, *Pseudomonas*, *Streptococcus*, and *yeast *	Culture negative	N/A
MSSA	MSSA	No
MSSA	MSSA	No
MSSA	MRSA	Yes
*Peptostreptococcus *	Deceased	N/A
Coagulase negative *Staphylococcus *	Enterobacter	Yes

^1^Eradicated: a failure due to reoperation where no evidence of recurrent infection was found.

^2^Methicillin-sensitive *Staphylococcus aureus*.

^3^Methicillin-resistant *Staphylococcus aureus*.

^4^Unknown: patient underwent rerevision elsewhere.

**Table 4:** Risk factors for failure: organisms.

	Fraction with failure, *n* (%)	RR	95% CI	*P* value
*Streptococcus *		3.18	2.13–4.72	<0.001
Yes	4/4 (100%)			
No	17/54 (32%)			

MSSA		1.26	0.63–2.53	0.510
Yes	8/19 (42%)			
No	13/39 (33%)			

MRSA		0.77	0.22–2.64	0.674
Yes	2/7 (29%)			
No	19/51 (37%)			

Coagulase negative *Staphylococcus *		0.88	0.42–1.84	0.737
Yes	7/21 (33%)			
No	14/37 (38%)			

Polymicrobial		3.31	2.19–5.00	<0.001
Yes	5/5 (100%)			
No	16/53 (30%)			

**Table 5:** Risk factors for failure: patient characteristics and surgical factors.

	Fraction with failure, *n* (%)	RR	95% CI	*P* value
Sex		1.15	0.56–2.35	0.707
Male	13/34 (38%)			
Female	8/24 (33%)			
ASA 3		0.96	0.45–2.08	0.927
Yes	6/17 (35%)			
No	15/41 (37%)			
Obese (>30 kg/m^2^)		0.89	0.41–1.92	0.761
Yes	10/29 (34%)			
No	7/18 (39%)			
Smoker		0.77	0.22–2.72	0.684
Yes	2/7 (29%)			
No	13/35 (37%)			
Soft tissue coverage (flap)		2.67	1.51–4.70	**0.001**
Yes	7/9 (78%)			
No	14/48 (29%)			
Spacer type		1.22	0.59–2.57	0.584
Block/all Cement mold	6/14 (43%)			
Articulating	15/43 (35%)			
≥4 surgeries between primary TKA and stage I		2.25	1.13–4.47	**0.020**
Yes	3/4 (75%)			
No	17/54 (33%)			
Type of infection		1.01	0.68–1.50	0.974
Early	6/18 (33%)			
Delayed	6/14 (43%)			
Late	9/26 (26%)			

**Table 6:** Failure and mortality rates reported in prior studies for two-stage revision TKA.

Reference (authors)	Year	Failure defined	Number of knees	Mean follow-up (months)	Failure rate (%)	Overall mortality rate (%)	90-day mortality rate (%)
Current study	2013	Persistent infection	66	40 (0–113)	24	23 (15/66)	1.5 (1/66)
Cai et al. [19]	2012	Reinfection	23	43 (24–60)	9	NR	NR
Kubista et al. [18]	2012	Reinfection	368	43 (1–93.6)	16	NR	NR
Mahmud et al. [20]	2012	SSI	253	12 (12–204)	13	NR	NR
Radoicic et al. [9]	2012	SSI	21	35–47	24	NR	NR
Tigani et al. [17]	2013	SSI	38	65 (24–139)	23.6	NR	NR
Gooding et al. [21]	2011	Reinfection	115	108 (60–144)	12	30 (32/115)	NR
Sherrell et al.^*^ [16]	2011	SSI	83	50 (1–151)	34	14 (12/83)	NR
Mortazavi et al. [14]	2011	SSI	117	41 (24–113)	28	NR	NR
Macheras et al. [22]	2011	Reinfection	34	145 (120–168)	9	NR	NR
Kurd et al. [23]	2010	SSI	96	35 (24–90)	27	NR	NR
Kosters et al. [24]	2009	Reinfection	6	25 (2–61)	0	NR	NR
Hofmann et al. [25]	2005	Reinfection	50	74 (24–150)	12	22 (11/50)	NR
Average					**17.3**	**22**	

SSI = subsequent surgery for reinfection; NR = not reported.

^*^Two-Stage after failed I&D.
